# Second-look flexible ureteroscopy after RIRS – Holmium Moses *versus* TFL (Soltive)

**DOI:** 10.25122/jml-2022-0180

**Published:** 2022-10

**Authors:** Bogdan Geavlete, Cristian Mareș, Răzvan Mulțescu, Dragoș Georgescu, Petrișor Geavlete

**Affiliations:** 1 Department of Urology, Sanador Hospital, Bucharest, Romania; 2 Department of Urology, Sf. Ioan Emergency Clinical Hospital, Bucharest, Romania

**Keywords:** retrograde intrarenal surgery, second-look flexible ureteroscopy, residual stone fragments

## Abstract

Retrograde intrarenal surgery (RIRS) is nowadays more and more indicated in pyelocaliceal stones. Holmium and Thulium lasers are the main lasers used. Fragmenting (basketing) or dusting, despite the new technology, still have residual stones (even having 250 microns). This study evaluates second-look flexible ureteroscopy for residual fragments. We analyzed 246 patients (October 2020–March 2022) on which we used Moses Holmium technology (187 cases) in Group 1 and Soltive Laser System (59 cases) in Group 2. The average stone size was 13.1 mm (range 11–29), and the average stone density was 1026 HU (range 870–1752). We used 270 µm for Ho: YAG laser and 150 µm for TFL. For Holmium, we applied energy 0.4J and frequency 80 Hz. For TFL, we applied fine dusting (0.15 J/100 Hz) and dusting (0.5 J/30 Hz). After three months, we practiced the second flexible ureteroscopy. Both groups were compared for completely visual stone-free rates. Stone-free rate at 3 months (second flexible ureteroscopy) was 86.63% (n=162/187) in Group 1 and 96,61% (n=57/59) in Group 2, respectively. The stone-free correlation with the CT preoperative evaluation was 160/187–85.56% for Holmium and 55/59–93.22% for TFL. We found residual stones after the second flexible ureteroscopy in 25 cases after Holmium laser treatment and only in 2 cases after TFL. In all these cases, we finally obtained zero residual stones. The second flexible ureteroscopy could achieve complete residual stone removal and real stone-free status. Despite a slight difference between these two laser technologies, the second look decreases the residual fragments.

## INTRODUCTION

Urolithiasis represents a common finding in the general population, affecting approximately 12% of the world population during their lifetime [1], with continuous increasing prevalence and recurrence rates yearly [2]. Although men are more affected than women, with the highest incidence between 20 and 50 years old, it can occur regardless of age and sex [3, 4]. Most urinary stones are located in the kidney, requiring different interventions. The introduction of the extracorporeal shock wave lithotripsy (ESWL) in the 1980s has revolutionized the modern minimally invasive stone treatment, and ever since, together with percutaneous nephrolithotomy (PCNL) has significantly decreased the need for open surgery while achieving stone-free rates of up to 80% [5, 6]. The continuous evolution and modernization of flexible endoscopes have promoted retrograde intrarenal surgery (RIRS) among the most frequent minimally-invasive procedure for most cases of kidney stones, according to both European [7] and American [8] guidelines on urolithiasis with promising results on stone-free rates [9, 10] and perioperative related morbidity [11].

The miniaturization of instruments and technological advances in image quality, maneuverability and laser lithotripsy systems have endorsed RIRS as a safe and efficient method of treatment for intrarenal stones [12, 13]. The actual gold standard of laser (light activation by the stimulated emission of radiation) technology in renal stone lithotripsy is HO: YAG (Holmium: Yttrium-Aluminum-Garnet) [14, 15], with three decades of demonstrating clinical potential and safeness in flexible endoscopic procedures [16]. New technology has emerged recently, with promising preliminary results on stone fragmentation and overall performance: Thulium fiber laser [17–19]. In mid-summer 2020, Olympus^TM^ launched the Solvite SuperPulsed Thulium Fiber Laser System for clinical application in endourology, with general availability and favorable results presented by several authors [20, 21].

The objective of laser lithotripsy in renal stones is to safely fragment stones into smaller parts (fragments-basketing) which can be easily extracted with various probes, or completely fragmenting the stones in "dust", which is defined as particles smaller than 250 µm on floating and sedimentation criteria and which can be easily evacuated on the ureteroscope's working channel, achieving the stone-free status [22, 23]. The latter was defined as no stones or residual stones of less than 2–4 mm on postoperative control KUB or non-contrast enhanced computed tomography [24, 25]. Different imaging techniques have been proposed to determine the success of stone fragmentation after various urological stone-related procedures, such as ultrasound, renal radiography and computed tomography. Recently, the idea of a second-look ureteroscopy procedure has emerged to evaluate the pyelocaliceal system for accurate visualization and certainty of an actual stone-free status [26, 27] and, if not already achieved, to serve as a supplementary curative technique in definitive stone treatment.

Considering the importance of achieving a good stone-free status, whenever it can be achieved, to decrease other post interventional stone-related events, this study aimed to determine the efficacy of a second-look ureteroscopy for a real analysis of residual fragments after dusting lithotripsy using both Holmium and Thulium laser fibers.

## Material and methods

**Figure 1:**
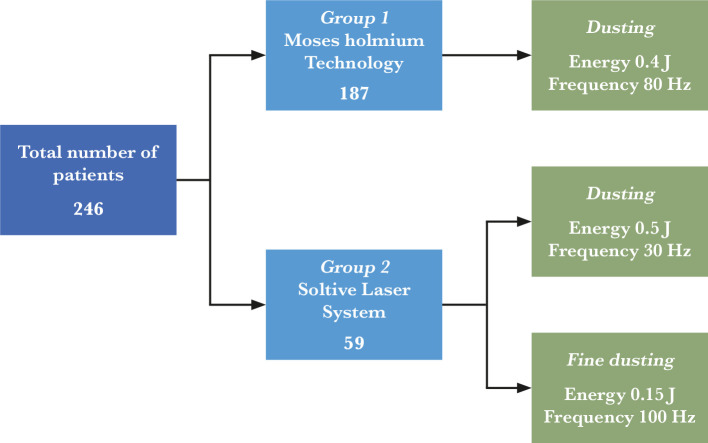
Group-patient diagram and modalities of treatment.

A retrospective analysis of 246 patients who underwent retrograde flexible ureteroscopy for pyelocaliceal stones was conducted between October 2020 and March 2022 at the Urology Department from Sf. Ioan Emergency Hospital in Bucharest, Romania. In 187 cases, Moses Holmium laser technology (distance mode) was used as laser lithotripsy (Group 1), while in 59 cases Soltive Laser system using Thulium laser fiber was the choice for stone dusting. In all cases, dusting and fine dusting were the pre-setting of frequency and energy of the laser systems. A detailed representative diagram of patients included in the study, along with the specific type of laser lithotripsy used for each group of patients, is represented in Figure 1.

In all cases, a CT urography (urography – CT) was conducted to evaluate the anatomy of the renourinary system and determine the stone density. The average stone size was 13.1 mm (ranging between 11–29 mm, measured as the longest dimension on imaging visualization) with a mean density of 1026 HU (range between 870–1752 HU).

The exclusion criteria were abnormalities of the renourinary system, incomplete data on stone type and loss of follow-up patients, cases where flexible ureteroscopy was performed by different surgeons and cases where incomplete stone dusting was performed due to intraoperative complications (*e.g*. bleeding followed by poor visualization).

All interventions were performed by a single, experienced surgeon with over 5000 flexible ureteroscopies performed on patients under general anesthesia. Sterile urine culture was mandatory in all cases before every procedure, and antibiotic prophylaxis was performed with a single-dose second-generation cephalosporin. All patients were previously catheterized with a Double J stent extracted at the beginning of the procedure. A ureteral access sheath was placed to facilitate access to the renal pelvis. All procedures were performed with reusable flexible ureteroscopes from Pussen (Pussen PU3033 A 7.5 Fr and Pussen 3022 9.5 Fr). In Group 1, the laser lithotripsy was performed with Moses Holmium technology, using a standard 270 µm laser fiber. In Group 2, the Soltive Laser System was used with a Thulium laser fiber of 150 µm. A Dormia basket probe was used to extract stones whenever larger fragments occurred. At the end of the procedure, a 6 Fr Double J stent was placed to avoid any obstructive postoperative complications due to residual stone fragments, steinstrasse or blood clots from bleeding. All the extracted stone fragments were sent for analysis for a complete metabolic evaluation and recurrence prevention.

The second-look flexible ureteroscopy was performed after 3 months to evaluate the pyelocaliceal system for residual stones. A CT scan was performed preoperatively for all symptomatic patients (renal colic, flank pain) to evaluate the possible residual stones. An auxiliary laser lithotripsy procedure was performed for any stone fragment larger than 1 mm to achieve a true stone-free status. In none of these cases, ureteral sheets were used, and no Double J stent was inserted. Each patient was informed about the procedures and other alternative surgical and/or medical treatment modalities. Patients chose the surgical approach after counseling with the surgeon.

Data were analyzed using Microsoft Excel software (Microsoft Corporation, Redmond, WA, USA). Simple descriptive statistics were calculated. The relationship of variables was analyzed using frequency and percentage.

## Results

A total of 246 patients were included in this study; demographic information for each selected patient, such as age, sex, body mass index (BMI), and location of the stone, was registered. The mean age was relatively similar in both groups Group 1 – 47.51 and Group 2 – 48.94, while the BMI value showed overweight patients in both groups, as follows Group 1 – 26.83 (range from 18 to 37), while in Group 2 – 25.05 (range from 20 to 37). A higher predilection was observed for male patients in both groups Group 1 – 56.14%, Group 2 – 54.23%, compared to female patients, Group 1 – 43.85%, Group 2 – 45.76%. The right-side location of stones was a more common finding, 57.75% and 69.49% respectively, compared to the left side (42.24% and 30.5%, respectively). More detailed information on the demographic characteristics of both groups is represented in Table 1.

**Table 1:** Demographic information of the studied groups.

	Age (years)		BMI (kg/m^2^)		Female		Male		Left side		Right side		Total	
	Mean	SD	Mean	SD	n	%	n	%	n	%	n	%	n	%
**Group 1**	47.51	14.72	26.83	3.32	82	43.85	105	56.14	79	42.24	108	57.75	187	76.01
**Group 2**	48.94	15.93	25.05	4.06	27	45.76	32	54.23	18	30.5	41	69.49	59	23.98
**Total**	47.85	15.02	26.90	3.29	109	44.3	137	55.69	97	39.43	149	60.56	-	-

The dimensions of stones were relatively similar in both of the selected groups, with a mean value of 13.1 mm. The stone density was slightly higher in the Thulium group, representing a mean value of 1045.1 HU, compared with the Holmium laser group, which accounted for a mean value of 1020.45 HU. An overall mean of 1026.36 HU in the entire study was determined. In all cases, small fragments or stone dust was sent for the biochemical study of stone composition. The most frequent lithiasis type was calcium oxalate, encountered in 148 (79.14%) cases in Group 1 and 41 (69.49%) in Group 2. It was followed by calcium phosphate: 28 (14.97%) in Group 1 and 15 (25.42%) in Group 2 and uric acid stones, which were encountered in 9 cases (4.81%) in Group 1 and 3 (5.08%) in Group 2. The least common stone type was cystine stones encountered in 2 cases (1.06%) in Group 1. A more detailed representation of stone-related clinical data is represented in Table 2.

**Table 2:** Stone-related clinical data.

	Stone characteristics				Stone composition							
	Stone size (mm)		Stone density (HU)		Calcium oxalate		Calcium phosphate		Uric acid		Cystine	
	Mean	SD	Mean	SD	n	%	n	%	n	%	n	%
**Group 1**	13.05	3.27	1020.45	110.85	148	79.14	28	14.97	9	4.81	2	1.06
**Group 2**	13.25	4.74	1045.1	109.19	41	69.49	15	25.42	3	5.08	-	-
**Total**	13.1	3.66	1026.36	110.73	189	76.82	43	17.47	12	4.87	2	0.81

The mean operative time was relatively similar in the Holmium laser Group 1 – 66.14 min, compared to the Thulium laser Group 2 – 64.06 min (Table 3). The overall mean operative time in the entire study was 65.65 min (range 45–90 min). Regarding postoperative complications, hematuria was the most frequent symptom encountered for 12 cases (6.41%) in Group 1 and 4 (6.77%) in Group 2. Other less frequent postoperative complications were noted as fever, which was encountered for 6 cases (3.2%) in Group 1 and 3 cases (5.08%) in Group 2 and renal colic, which was encountered for only 1 case (0.53%) in Group 1. All data presented were registered for the first main intervention; the mean operative time was much lower in the second-look RIRS, and no postoperative events were encountered after the second intervention. All postoperative complications after the first intervention were graded as Grade 1 or Grade 2 on Clavien-Dindo Classification System and were treated conservatory for a maximum of 3 days; no additional surgical or endoscopic procedures were required.

**Table 3:** Operative time and perioperative events.

	Operative time (min)		Postoperative events					
			Hematuria		Fever		Renal colic	
	Mean	SD	n	%	n	%	n	%
**Group 1**	66.14	13.62	12	6.41	6	3.2	1	0.53
**Group 2**	64.06	15.01	4	6.77	3	5.08	-	-
**Total**	65.65	13.96	16	6.5	8	3.25	1	0.4

**Figure 2:**
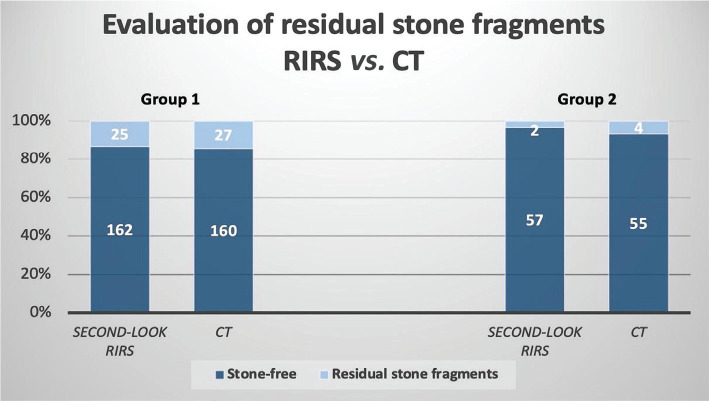
Comparing stone-free rates and residual fragments evaluation by flexible ureteroscopy and computer tomography in both groups (*RIRS – retrograde intrarenal surgery; CT – computer tomography).

All symptomatic patients underwent a non-contrast-enhanced CT to evaluate the residual fragments after the first intervention. After 3 months, a second-look flexible ureteroscopy was performed to assess the "true" stone-free status. In Group 1 (holmium laser), imaging investigations determined 160 cases out of 187 as stone-free (85.56%). In contrast, in Group 2 (Thulium laser), 55 of the 59 patients were considered stone-free (93.22%). Subsequently, all of these patients underwent a second flexible ureteroscopy which revealed better "real stone-free" status than imaging investigations. Thus, in Group 1, out of 187 patients, 162 were diagnosed as stone-free visually, representing 86.63%, while in Group 2 – 57 of the 59 patients did not show any residual fragment (96.61%) after the second intervention, highlighting a better evaluation of the true stone-free status after a close visualization of the pyelocaliceal system using flexible ureteroscopy. A visual diagram of both groups comparing stone-free rates and residual fragments by both imaging and interventional evaluation is represented in Figure 2.

## Discussion

Nephrolithiasis is a severe, recurrent condition that affects up to 10% of the adult population in the European Union annually [28]. The European Association of Urology recommends RIRS as a first-line treatment in kidney stones with dimensions between 1 and 2 cm, representing a significant volume of cases [7]. Laser technology has revolutionized the lithotripsy of kidney stones, representing the standard management in flexible ureteroscopy, with high-performance Holmium-type lasers able to exceed the recommended size and operate large kidney stones with good patient results [29]. Furthermore, the introduction of the Thulium laser in modern endourology seems to further revolutionize the management of kidney stones with superior results regarding stone dusting and postoperative stone-free status. The results of this study are similar to the data in the literature, obtaining over 90% stone-free rates in the second group, the one that uses the Thulium laser and over 85% in the first group, corresponding to the Holmium laser [30, 31].

Demographic factors play an important role in the development and evolution of kidney stones. The mean age of the incidence of kidney stones in the present study group was around 50 years of age, probably considering the known risk factors that occur, such as dietary factors, recurrent urinary tract infections and body weight; the effect of aging on lithiasis has been studied [32]. Most patients who presented stones in the study group were overweight, averaging approximately 27 kg/m^2^. Studies in obese patients with urinary stones have shown variable risk factors, such as urinary pH, systemic inflammation and oxidative stress being the primary places as having a negative influence on the prevalence of this pathology [33]. In the present study, both groups determined the highest incidence of kidney stones among the male population (55.7% *vs*. 44.3%). Recent studies show a changing trend in the incidence of lithiasis in relation to gender, with a significant increase in this disease among the female population, especially during adolescence [34]. Also, the risk of developing chronic kidney disease (CKD) is higher for female patients, so this pathology must be treated with higher caution [35].

The size of the calculus is a very important aspect of obtaining a stone-free outcome. With the advancement of laser lithotripsy technology in flexible ureteroscopy, the indication of stone dusting of voluminous stones acquires a more accentuated contour. The average size of the stones in the current study was 13.1 mm. Data from the literature suggest the possibility of managing large stones or difficult cases with promising results in the stone-free rates of the patient after the intervention [36–39] and even performing flexible ureteroscopy for staghorn stones in selected cases [40, 41]. In the present study, the mean density of the stones (measured in Hounsfield units at the CT examination) was 1026.36 HU. The most common type of stone was calcium oxalate (76.8%), followed by calcium phosphate (17.47%), uric acid (4.87%) and cystine (0.81%). A large study in the United States showed similar rates in the prevalence of matrix types that make up kidney stones, with a major predominance of calcium oxalate monohydrate and calcium phosphate, accounting for over 93% of their components [42].

The average operative time was 65 minutes, and the postoperative incidents were mostly represented by hematuria, fever and renal colic. These complications are among the most common in this endourological procedure, with data from the literature showing relatively similar prevalence rates [43, 44]. There were no major complications, such as perforation or ureteral avulsion, significant bleeding that required blood transfusion or sepsis in the postoperative period, these incidents being known to be among the most serious complications [45].

Computed tomography (CT) is the best imaging investigation for diagnosing and following patients with kidney stones. The sensitivity and specificity of this radiological investigation are approximately 95%. In a few cases, especially when the size of the stones is <3 mm, they can escape radiological detection and cannot be observed by this investigation [46]. Except for a few cases, such as indinavir stones or matrix proteases, all types of stones are seen on CT [47]. However, there are exceptional situations when various pathologies associated with the kidney can be confused with kidney stones. These elements of confusion with urinary stones include Randall's plaque, calcifications of the papilla or renal mucosa, or isolated cases of nephrocalcinosis [48]. These elements of calcification may be a reason for misinterpretation of spiral CT images in a patient known to have kidney stones and who is being monitored after surgery. The CT evaluation of the operated patients in this study showed a small number of patients – 2 cases in Group 1 and 2 cases in Group 2 in which the CT examination revealed the existence of post interventional residual lithiasis. Still, subsequent endoscopic investigation denied their presence during the extensive and thorough pyelocalicoscopy examination.

An important limitation of this study is the small number of patients. A higher number of patients could prove an even greater significance of the second look ureteroscopy or, in turn, disprove this percentage of false-positive imaging results in the diagnosis of residual kidney stones. Another limitation is that the study was developed in a single center, and a single experienced surgeon performed all interventions. These premises may represent the pioneer of a larger future study involving more patients from different centers and interventions performed by surgeons with variable experience for better results and to draw definitive conclusions.

## Conclusion

Following the consistent assessment of all patients after flexible ureteroscopy by systematic CT evaluations of endoscopic control and re-evaluation of the pyelocaliceal system, this study demonstrates that direct, optical and modern endoscopic visualization is superior to imaging investigations in detecting residual fragments after stone lithotripsy. This is the only feasible tool to determine the "true" stone-free rate condition after endourological interventions of urinary stones.
